# Integrated mental healthcare and vocational rehabilitation for people on sick leave with stress-related disorders: 24-month follow-up of the randomized IBBIS trial

**DOI:** 10.5271/sjweh.4084

**Published:** 2023-05-01

**Authors:** Andreas Hoff, Rie Mandrup Poulsen, Jonas Peter Fisker, Carsten Hjorthøj, Merete Nordentoft, Anders Bo Bojesen, Lene Falgaard Eplov

**Affiliations:** 1 Copenhagen Research Centre for Mental Health – CORE, Mental Health Centre Copenhagen, Copenhagen University Hospital, Hellerup, Denmark.; 2 The National Board of Social Services in Denmark, Odense, Denmark.; 3 Hejmdal Private hospital, Frederiksberg, Denmark.; 4 University of Copenhagen, Department of Public Health, Section of Epidemiology, Copenhagen, Denmark.; 5 Department of Public Health, Section of Social Medicine, University of Copenhagen, Copenhagen, Denmark.

**Keywords:** adjustment disorder, common mental disorder, distress, exhaustion disorder, integrated service, randomized trial, return to work, return-to-work, stepped care

## Abstract

**Objectives:**

Integrating vocational rehabilitation and mental healthcare has shown effects on vocational outcomes during sick leave with common mental disorders. In a previous paper, we showed that a Danish integrated healthcare and vocational rehabilitation intervention (INT) had a surprisingly negative impact on vocational outcomes compared to service as usual (SAU) at 6- and 12-month follow-up. That was also the case with a mental healthcare intervention (MHC) tested in the same study. This article reports the 24-month follow-up results of that same study.

**Method:**

A randomized, parallel-group, three-arm, multi-centre superiority trial was conducted to test the effectiveness of INT and MHC compared to SAU.

**Results:**

In total, 631 persons were randomized. Contrary to our hypothesis, SAU showed faster return to work than both INT [hazard rate (HR) 1.39, P=0.0027] and MHC (HR 1.30, P=0.013) at 24-month follow-up. Overall, no differences were observed regarding mental health and functional level. Compared to SAU, we observed some health benefits of MHC, but not INT, at 6-month follow-up but not thereafter, and lower rates of employment at all follow-ups. Since implementation problems might explain the results of INT, we cannot conclude that INT is no better that SAU. The MHC intervention was implemented with good fidelity and did not improve return to work.

**Conclusion:**

This trial does not support the hypothesis that INT lead to faster return to work. However, implementation failure may explain the negative results.

Long-term sick leave is a risk factor for exclusion from the labor market (1) and return to work (RTW) is associated with better health (2). Stress-related disorders account for a large part of sick leave (3). Consequently, much research has focused on developing and studying interventions to improve vocational outcomes after sick leave. A 2018 review showed that effective interventions usually consist of several components (4), and studies have hypothesized that these components should be integrated (5). Therefore, we conducted a trial (6, 7) to test the Integrated Healthcare and Vocational Rehabilitation (IBBIS) intervention (hereafter referred to as INT). INT aimed to improve both vocational and health outcomes, and we compared it to service as usual (SAU) as well as a mental healthcare intervention (MHC). The primary outcome was time to stable RTW at 12-month follow-up. All results from 6- and 12-months follow-up are published (7). Contrary to our initial hypothesis, SAU showed considerably faster RTW compared to INT [hazard rate (HR) 1.43, P=0.002]. However, INT showed some benefits on some exploratory outcomes but not on secondary outcomes. Compared to MHC, SAU similarly caused increased RTW (HR 1.35, P=0.008) but lower self-reported scores on levels of symptoms and functioning. Thus, against expectations, SAU did not clearly imply insufficient healthcare treatment, and that was the main reason for comparing INT not only to SAU but also to MHC. Conclusively, INT was found to be inferior overall, while MHC was only partially inferior because of the observed the health benefits. This paper reports the 24-month follow-up, since we hypothesized that INT would also yield long-term sustainable RTW that persisted beyond the intervention period.

## Methods

Methods are reported elsewhere (6, 7) (supplementary material, www.sjweh.fi/article/4084, supplement 1). Eligible participants were adult sickness absentees (≥4 weeks), with a stress-related disorder. One outcome, recurrence of sick leave, was added at 24 months only. In INT, participants received best practice mental healthcare (BP-MH) and IBBIS vocational rehabilitation (IBBIS-VR). BP-MH was a stepped-care intervention, with treatment intensity determined by baseline symptom level. IBBIS-VR was based on the principles of the sharp-at work-intervention (8) and individual placement and support (9). The INT components were integrated through a range of activities using relational coordination (10). In SAU, participants received any primary sector healthcare and municipal VR they would otherwise have received, had they not been randomized. In MHC, participants received BP-MH but VR in municipal facilities, as in SAU. In INT and MHC, the practitioners’ adherence to manuals was examined through fidelity reviews. Data were obtained through self-report and registers. Outcomes at 24 months were time-to-RTW (a secondary outcome), weeks in work, proportion in work and a range of self-reported outcomes measures, including symptoms, all of which are described in supplement 1. Post-hoc, for 24-month follow-up only, we decided to count the crude number of recurrent sick leaves but without using statistical parametrization.

### Statistical analyses

Proportion-in-work outcomes were analyzed using logistic regression and time-to-RTW outcomes using Cox-regression. Self-reported outcomes were analyzed using linear mixed-effects models. Throughout, we adhered to the intention-to-treat principle. Due to multiple testing, P-values <0.017 were considered statistically significant and those <0.05 borderline (the latter was a post-hoc decision). Subgroup analyses were performed according to selected baseline values and time. Sensitivity analyses were conducted by imputing all missing data in best/worst case scenarios, and regarding return to stable work outcomes with different thresholds for what minimal duration constituted “stable” RTW (1, 4, 8 or 12 weeks).

## Results

**Figure 1:**
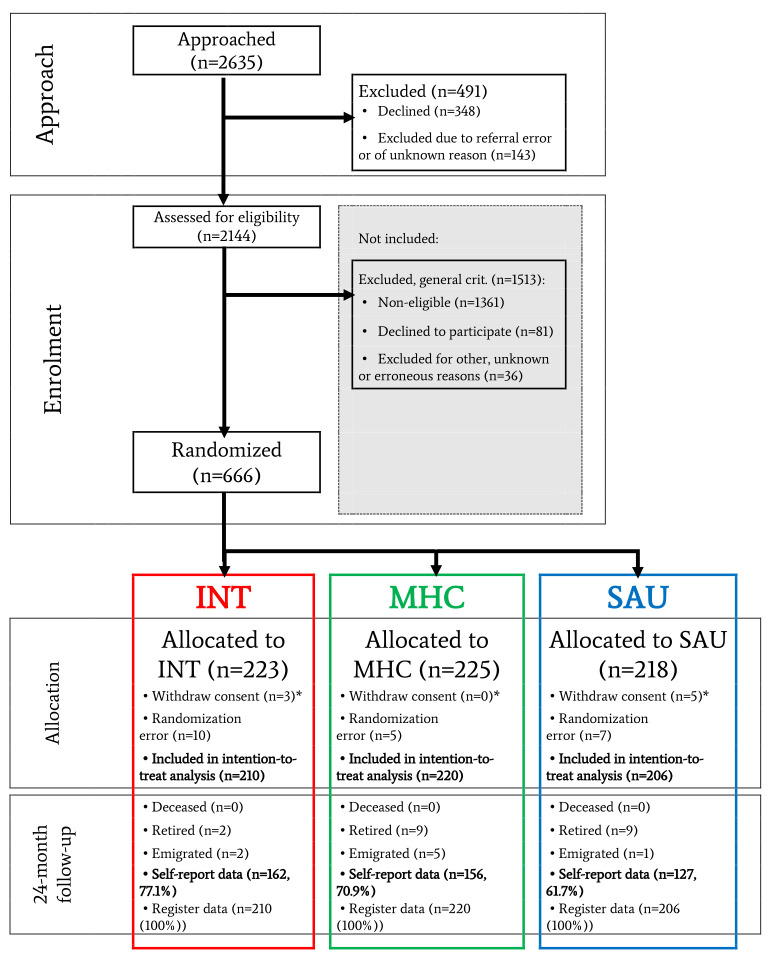
Participant flow. [INT=IBBIS integrated intervention; MHC=mental healthcare; SAU=service as usual; RCT=randomized controlled trial]. *Due to European data legislation, complete deletion of data was possible and was requested after randomization by 8 participants.

**Figure 2:**
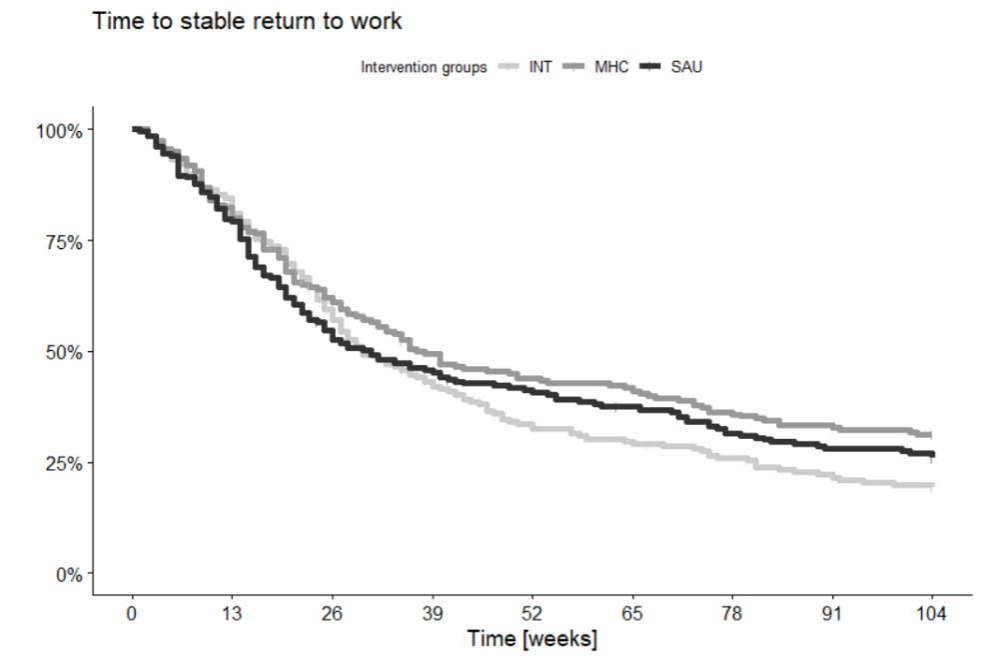
Kaplan-Meier curve for time to the event ‘first stable return to work’. [INT=IBBIS integrated intervention; MHC=mental healthcare; SAU=service as usual.

Analyses included 636 participants in total. Figure 1 depicts participant flow, while table 1 shows baseline characteristics. SAU showed faster RTW rates than both MHC with a HR of 1.30 (P=0.013) and INT with a HR of 1.39 (P=0.003). We did not observe a similar difference between INT and MHC. SAU showed a significantly higher number of weeks in work than both MHC with a rate ratio (RR) of 1.21 (P=0.003) and INT with an RR of 1.16 (P=0.016), but no differences were detected between MHC and INT. Proportions in work were similar across the groups at 24-month follow-up. Figure 2 displays the Kaplan-Meier curve for the three groups and table 2 the results of all other vocational outcomes. Two years after baseline, the level of anxiety was slightly higher in the SAU group compared to the MHC group, with a borderline statistically significant mean difference of 1.58 (P=0.04). No other self-reported differences were seen at 24-month follow-up. Recurring sick leave and all self-reported outcomes are presented in supplement 2. Sensitivity analyses of all outcomes are shown in supplement 3 and subgroup analyses for all outcomes in supplement 4. We detected no statistically significant interaction or subgroup deviations. Fidelity reviews showed that BP-MH was implemented with high fidelity but IBBIS-VR with only fair fidelity; see supplement 5.

**Table 1:** Baseline characteristics. [SD=standard deviation; MHC=mental healthcare; SAU=service as usual; INT=integrated intervention.]

		Groups							
		INT (N=210)			MHC (N=220)			SAU (N=206)	
		N (%)	Mean (SD)		N (%)	Mean (SD)		N (%)	Mean (SD)
Age (years)			45.22 (9.42)			45.26 (10.08)			43.18 (10.23)
Gender									
	Female	155 (73.8)			163 (74.1)			169 (82.0)	
	Male	55 (26.2)			57 (25.9)			37 (18.0)	
Educational level (highest level achieved									
	Primary school	18 (8.6)			16 (7.3)			15 (7.3)	
	Secondary / vocational	61 (29)			68 (30.9)			62 (30.1)	
	Bachelor (professional) and academic degree (master level)	131 (62.4)			136 (61.8)			129 (62.6)	
Municipality									
	Copenhagen	127 (60.5)			133 (60.5)			121 (58.7)	
	Gentofte	26 (12.4)			27 (12.3)			28 (13.6)	
	Gladsaxe	29 (13.8)			30 (13.6)			28 (13.6)	
	LTK Lyngby-Taarbæk	28 (13.3)			30 (13.6)			29 (14.1)	
Vocational status									
Employed		177 (84.3)			188 (85.5)			177 (85.9)	
Unemployed		33 (15.7)			32 (14.5)			29 (14.1)	
Bech Depression Inventory ^a^			21.15 (8.45)			20.45 (8.88)			20.35 (8.23)
Bech Anxiety Inventory ^a^			15.70 (8.76)			14.43 (7.56)			15.75 (7.49)
Perceived Stress Scale ^a^			23.32 (5.48)			22.67 (5.52)			23.13 (5.53)
Work and Social Adjustment Scale ^b^			21.46 (7.92)			20.97 (7.93)			22.05 (7.92)
Sick leave (weeks)			10.62 (2.80)			11.06 (3.99)			11.34 (3.66)
Diagnosis									
	Adjustment disorder	41 (19.5)			37 (16.8)			39 (18.9)	
	Exhaustion disorder	109 (51.9)			118 (53.6)			108 (52.4)	
	Stress	60 (28.6)			65 (29.5)			59 (28.6)	

^a^ % missing=0.3 ^b^ % missing=2.2

**Table 2a:** Vocational outcomes at 24-month follow-up: *group values.* [MHC=mental healthcare; SAU=service as usual; INT=IBBIS integrated intervention; IQR=interquartile range; CI=confidence interval.]

	Group values											
	N	INT				MHC				SAU		
		Weeks (IQR)	%	Mean (SD)		Weeks (IQR)	%	Mean (SD)		Weeks (IQR)	%	Mean (SD)
Time to return to work	636	25 (16–86)				23 (14–70)				19 (11–43)		
Proportion in work	636		58.2				55.5				60.5	
Weeks of work	616			48.4 (11.4)				47.2 (10.6)				57.5 (12.7)

**Table 2b:** Vocational outcomes at 24-month follow-up: *pairwise comparisons*. [MHC=mental healthcare; SAU=service as usual; INT=IBBIS integrated intervention; IQR=interquartile range; CI=confidence interval.]

	Group comparisons									
	N	SAU - MHC			SAU - INT			MHC - INT		
		Ratio (98.3% CI)	P-value		Ratio (98.3% CI)	P-value		Ratio (98.3% CI)		P-value
Time to return to work	636	1.3 ^a^ (1.01—1.69)	*0.013		1.39 ^a^ (1.07—1.81)	*0.0027		1.07 ^a^ (0.82—1.39)		0.527
Proportion in work	636	1.21 ^b^ (0.74—1.98)	0.342		1.07 ^b^ (0.64—1.76)	0.762		0.88 ^b^ (0.54—1.45)		0.547
Weeks of work	616	1.21 ^c^ (1.04—1.42)	*0.0029		1.16 ^c^ (1— 1.35)	*0.016		0.97 ^c^ (0.82—1.14)		0.605

^a^ Hazard ratio. ^b^ Odds ratio. ^c^ Rate ratio. * P<0.0167.

## Discussion

SAU yielded consistently better vocational outcomes across multiple measures at all follow-ups compared to both INT and MHC. Regarding symptoms, the slightly lower level of anxiety (borderline statistical significance) in the MHC group compared to SAU at 24-month follow-up might be a chance finding since no other differences were seen across many symptom scales. Both MHC and INT failed to consistently improve the RTW process as hypothesized. Possible explanations for this failure include implementation, programme, or methodological failure (11).

*Indications of implementation failure (the consequence of suboptimal implementation of an otherwise truly beneficial intervention):* Through fidelity reviews, we observed a range of implementation issues: IBBIS-VR and the activities integrating the two intervention components showed lower implementation degree. As these two parts of the intervention were only part of INT, we can conclude that only interventions in the MHC group were implemented with high fidelity. True integration of the intervention components in INT was challenged by lack of trust and diverging norms and goals between the different sectors, as described elsewhere (12, 13). In other studies, VR services similar to IBBIS-VR have shown positive effect. BP-MH in the INT and MHC groups was implemented with high fidelity. Overall, these observations suggest that the negative outcome of INT may be due to implementation failure.

*Indications of theory failure (when an intervention is not beneficial for the participants, even if the intervention is delivered perfectly as protocolled):* Theory failure is indicated because MHC interventions were sufficiently implemented. Since BP-MH was delivered in INT as well, part of the failure of INT may also be due to theory failure.

*Indications of methodological failure (when an otherwise truly beneficial intervention fails to show positive outcome due to invalid outcome measures):* While time-to-RTW is the most common outcome measure in this research field, there is no consensus regarding specific definitions and measures of RTW (14). Strictly defined, the measure implies that the most optimal RTW time is zero. We chose RTW as the primary outcome measure on the basis of scientific precedence, but we argue that this outcome may not be the most appropriate since *too fast* RTW could imply premature exposure to workplace risk factors for relapse.

### Limitations

Implementation issues severely limit both the internal and external validity of the study. Furthermore, due to the nature of the interventions, participants could not be blinded, and the study may be limited by some effects of the allocation procedure in itself.

### Implications

Complex interventions involving co-location and integration of multidisciplinary teams are generally difficult to implement (15), as demonstrated by our study and other studies. Even if the interventions are truly beneficial, and the failure to show positive effects in this study solely rests on poor implementation, integration of services requires considerable management and administration. Therefore, any stakeholder intending to either trial or practice similar interventions should be duly cautioned and advised to pilot interventions more elaborately before large scale trialing. Furthermore, any effect of integration activities in our study is probably moderated by national service legislation and properties of the organization that the study intervention in embedded within (16). Thus, international generalization of these effects may not be feasible. Regarding choice of outcomes, further research and discussions between relevant stakeholders should take place to elucidate which outcome set best captures the quality of an individual’s RTW process.

### Concluding remarks

This trial compared INT and MHC to SAU. Contrary to our initial hypothesis, both INT and MHC consistently showed significantly lower RTW rates across all follow-ups, compared with SAU. MHC yielded some short-term health benefits, but they were not sustained beyond six months. However, as we cannot rule out implementation failure in INT, and because BP-MH may not have constituted an equally or more qualified mental health service than SAU, we cannot conclude solidly on the results.

### Ethics

The trial was registered at www.clinicaltrials.gov (#NCT02885519) and evaluated and approved by the Regional Ethics Committees of the Capital Region (# H-16015724) and the Danish Data Protection Agency (#RHP-2016-006). It was conducted in accordance with Danish and European regulations. An IBBIS team member informed every participant about the objective of the study and the implications of participation, and all participants gave oral and written consent before enrolment.

## Supplementary material

Supplementary material

## References

[1] Hultin H, Lindholm C, Möller J. Is there an association between long-term sick leave and disability pension and unemployment beyond the effect of health status?--a cohort study. PLoS One 2012;7(4):e35614. 10.1371/journal.pone.0035614 22558176 PMC3338415

[2] Rueda S, Chambers L, Wilson M, Mustard C, Rourke SB, Bayoumi A, et al. Association of Returning to Work With Better Health in Working-Aged Adults: A Systematic Review. Am J public Heal. 2012;102(3):541–56. 10.2105/AJPH.2011.300401 PMC3487667 22390520

[3] Søgaard HJ, Bech P. Psychiatric disorders in long-term sickness absence -- a population-based cross-sectional study. Scand J Public Health 2009 Sep;37(7):682–9. 10.1177/1403494809344357 19700479

[4] Mikkelsen MB, Rosholm M. Systematic review and meta-analysis of interventions aimed at enhancing return to work for sick-listed workers with common mental disorders, stress-related disorders, somatoform disorders and personality disorders. Occup Environ Med 2018 Sep;75(9):675–86. 10.1136/oemed-2018-105073 29954920

[5] OECD, The Organization for Economic coordination and Development. Sick on the Job? Myths and Realities about Mental Health and Work. 2012.

[6] Poulsen R, Hoff A, Fisker J, Hjorthøj C, Eplov LF. Integrated mental health care and vocational rehabilitation to improve return to work rates for people on sick leave because of depression and anxiety (the Danish IBBIS trial): study protocol for a randomized controlled trial. Trials 2017 Dec;18(1):578. 10.1186/s13063-017-2272-1 29197414 PMC5712198

[7] Hoff A, Fisker J, Poulsen RM, Hjorthøj C, Rosenberg NK, Nordentoft M et al. Integrating vocational rehabilitation and mental healthcare to improve the return-to-work process for people on sick leave with stress-related disorders: results from a randomized trial. Scand J Work Environ Health 2022 Jul;48(5):361–71. 10.5271/sjweh.4021 35373306 PMC9527782

[8] Arends I, van der Klink JJ, van Rhenen W, de Boer MR, Bültmann U. Prevention of recurrent sickness absence in workers with common mental disorders: results of a cluster-randomised controlled trial. Occup Environ Med 2014 Jan;71(1):21–9. 10.1136/oemed-2013-101412 24158311

[9] Modini M, Joyce S, Mykletun A, Christensen H, Bryant RA, Mitchell PB et al. The mental health benefits of employment: results of a systematic meta-review. Australas Psychiatry 2016 Aug;24(4):331–6. 10.1177/1039856215618523 26773063

[10] Gittell JH. Relational coordination: Coordinating work through relationships of shared goals, shared knowledge and mutual respect. In: Kyriakidou O, Èzbilgin M, editors. Relational Perspectives in Organizational Studies: A Research Companion. Sheltenham, USA; 2006. p. 74–94.

[11] Stame N. What Doesn’t Work? Three Failures, Many Answers. Eval (London, England 1995). 2010;16(4):371–87.

[12] Poulsen RM, Pii KH, Bültmann U, Meijer M, Eplov LF, Albertsen K et al. Developing Normative Integration among Professionals in an Intersectoral Collaboration: A Multi-Method Investigation of an Integrated Intervention for People on Sick Leave Due to Common Mental Disorders. Int J Integr Care 2019 Nov;19(4):4. 10.5334/ijic.4694 31749668 PMC6838772

[13] Poulsen RM, Pii KH, Eplov LF, Meijer M, Bültmann U, Christensen U. Developing Interpersonal Trust Between Service Users and Professionals in Integrated Services: Compensating for Latent Distrust, Vulnerabilities and Uncertainty Shaped by Organisational Context. Int J Integr Care [Internet]. 2021 [cited 2022 Mar 18];21(3). Available from: /pmc/articles/PMC8252972/. 10.5334/ijic.5599 PMC8252972 34248445

[14] Hoving J, Ravinskaya M, Verbeek J, Langendam M, Kunz R, Verstappen S et al. The reporting of work participation outcomes and measurement methods in randomized controlled trials: a systematic review. Saf Health Work 2022;13:S103–103. 10.1016/j.shaw.2021.12.1076 34715311

[15] Bonfils IS, Hansen H, Dalum HS, Eplov LF. Implementation of the individual placement and support approach–facilitators and barriers. Scand J Disabil Res 2017;19(4):318–33. 10.1080/15017419.2016.1222306

[16] MacEachen E, Varatharajan S, Du B, Bartel E, Ekberg K. The Uneven Foci of Work Disability Research Across Cause-based and Comprehensive Social Security Systems. Int J Health Serv 2019 Jan;49(1):142–64. 10.1177/0020731418809857 30428268

